# Quantitative Evaluation of 3D Mouse Behaviors and Motor Function in the Open-Field after Spinal Cord Injury Using Markerless Motion Tracking

**DOI:** 10.1371/journal.pone.0074536

**Published:** 2013-09-18

**Authors:** Alison L. Sheets, Po-Lun Lai, Lesley C. Fisher, D. Michele Basso

**Affiliations:** 1 Department of Mechanical and Aeronautical Engineering, The Ohio State University, Columbus, Ohio, United States of America; 2 Civil, Environmental and Geodetic Engineering, The Ohio State University, Columbus, Ohio, United States of America; 3 School of Health and Rehabilitation Sciences, The Ohio State University, Columbus, Ohio, United States of America; Hospital Nacional de Parapléjicos, Spain

## Abstract

Thousands of scientists strive to identify cellular mechanisms that could lead to breakthroughs in developing ameliorative treatments for debilitating neural and muscular conditions such as spinal cord injury (SCI). Most studies use rodent models to test hypotheses, and these are all limited by the methods available to evaluate animal motor function. This study’s goal was to develop a behavioral and locomotor assessment system in a murine model of SCI that enables quantitative kinematic measurements to be made automatically in the open-field by applying markerless motion tracking approaches. Three-dimensional movements of eight naïve, five mild, five moderate, and four severe SCI mice were recorded using 10 cameras (100 Hz). Background subtraction was used in each video frame to identify the animal’s silhouette, and the 3D shape at each time was reconstructed using shape-from-silhouette. The reconstructed volume was divided into front and back halves using k-means clustering. The animal’s front Center of Volume (CoV) height and whole-body CoV speed were calculated and used to automatically classify animal behaviors including directed locomotion, exploratory locomotion, meandering, standing, and rearing. More detailed analyses of CoV height, speed, and lateral deviation during directed locomotion revealed behavioral differences and functional impairments in animals with mild, moderate, and severe SCI when compared with naïve animals. Naïve animals displayed the widest variety of behaviors including rearing and crossing the center of the open-field, the fastest speeds, and tallest rear CoV heights. SCI reduced the range of behaviors, and decreased speed (r = .70 p<.005) and rear CoV height (r = .65 p<.01) were significantly correlated with greater lesion size. This markerless tracking approach is a first step toward fundamentally changing how rodent movement studies are conducted. By providing scientists with sensitive, quantitative measurement methods, subjectivity and human error is reduced, potentially providing insights leading to breakthroughs in treating human disease.

## Introduction

Development of new, effective treatments for debilitating neural and muscular conditions such as spinal cord injury (SCI) depends on identifying cellular targets. Testing of potential treatments and cellular hypotheses commonly occur in rodent models of these devastating conditions. Thus, these studies require accurate, quantitative and sensitive measures of rat or mouse motor function. While a variety of specialized behavioral tests have been developed, such as electromyography (EMG) [1], [2], reflex testing [3], and swimming [4]–[7], few have gained wide-spread use in mice with SCI. Three of the more common assessments use visual observation of behavior in an open-field [8], [9], kinematic and footprint analysis of constrained walking on a treadmill or elevated walkway [1], [10], [11], or breaking beams of infrared light to measure activity [12]. While these approaches have strengths, their limitations may preclude further advancements in the development of effective treatments.

Open-field testing of mice and rats enables evaluation of differences in behavioral preferences as well as motor function ranging from complete paralysis to normal locomotion, but subjective scoring reduces measurement precision [8], [9]. The Basso Mouse Scale for Locomotion (BMS) [9] is a widely-used, validated, semi-quantitative rating scale for open-field locomotion in spinal cord injured mice. It is based on visual observations of individual animals moving freely in a 36 in diameter, round environment. Injury severity is rated by pairs of highly trained researchers on a 9-point scale that quantifies visually detectable locomotion events over a 4-minute testing period. Locomotion events include evaluation of forelimb and hindlimb coordination during sustained locomotion, trunk instability, paw orientation, and tail position among others [9]. This rating system is reliable and repeatable, but movements in mice are extremely small and fast so multiple observations of events during the 4-min testing period are needed and researchers must work in teams. Additionally, events and behaviors must be visually detectable, which potentially excludes important gait characteristics that may indicate recovery, such as the spatial distribution of movements and speed.

Kinematics provide greater sensitivity than visually-based assessments [13] but cannot be used in non-stepping rodents. Marker-based methods are used to quantify mouse limb kinematics while walking on treadmills or over-ground on straight walkways, but there are questions about the accuracy and biological relevance of these approaches. Relative motion between the skin and underlying bones affects the accuracy of marker-based measurements of rodents more than humans [14]. In our experience, marker placement over boney landmarks must be performed while the rodent is standing or walking in order to minimize the effect of skin movement. The challenges of accurate marker placement are compounded when rodents remove them during the data collection. Additionally, marker positions during the trial are often hand-digitized which is a considerable time burden and limits the number of groups, rodents and evaluations in experimental designs. While strategies to limit these weaknesses have been developed [5], [15], [16], they never the less remain a large source of error. Lastly, to our knowledge, kinematics have only been applied to analyze constrained walkway or treadmill locomotion and not to movements in the open field. In these situations, the animal can only walk in a straight line, and is typically required to move at a constant speed. It is unknown whether locomotion patterns are the same in open-field environments as during constrained 2D movements. For example, differences in stride frequency and length have been found for mice during treadmill and over-ground walking [17]. It is possible that measurements of a wide variety of 3D movements in the open-field would exaggerate deficiencies in injured animals, and could provide more biologically relevant data that allows finer scale evaluations of injury severity and recovery.

Approaches that enable measurement in small open-field environments have used infrared light to monitor movement type or a single video stream to measure foot placement, but both have limitations. Activity monitors automatically calculate movement time, distance, and type as rodents move in a small open-field, but they cannot differentiate the quality of ambulatory movements (i.e. stepping vs. dragging). A video-based approach that enabled automated analysis of gait/footprint measurements in an open-field has been proposed [18], [19]. Image processing techniques were used to measure locations of the mouse’s paws, and then automatically output measures of forelimb and hindlimb placement and coordination similar to those reported from straight walkway and treadmill studies (ex. CatWalk by Noldus Information Technology, TreadScan by Clever Sys Incorporated, and DigiGait by Mouse Specifics, Inc). This approach was limited to only evaluating animals capable of stepping in a small open-field environment (53×34.5 cm rectangle), and no behavioral measures were developed [18], [19].

An ideal movement analysis system would combine the strengths of all previous systems and enable quantitative kinematics to be collected automatically in the open-field environment for animals with motor function ranging from paralysis to walking. Applying three-dimensional (3D) computer vision techniques to measure mouse behavior in the open-field environment may enable the development of quantitative and sensitive measures of mouse locomotor capabilities. Reconstructing an object’s 3D shape from multiple silhouettes [20] has been successfully applied to human body shape [21] and to track human movements over time [22]–[24], but few studies have applied this approach to evaluate animal movement [25], [26]. The advantage of the shape-from-silhouette approach is that it allows parameters to be calculated such as center of volume (CoV) position and overall animal shape. Since tissue densities in humans and animals are roughly evenly distributed left-to-right and top-to-bottom, CoV position can approximate the center of mass. Center of mass positions in humans reflect gross movement patterns of locomotion speed, step length and energy efficiency [27]–[29]. It is likely that center of mass, and thus CoV, information for mice would be equally descriptive of gross locomotion patterns.

An early step in developing a new SCI recovery measure is establishing validity. Predictive and concurrent validity are especially relevant for SCI because they test whether the new measure predicts the severity of the injury and if it produces similar results as well-accepted measures collected concurrently. Until validity is proven, the evaluation of other psychometric variables such as reliability, sensitivity and generalizability are premature.

The purpose of this study was to develop an automatic behavioral and locomotor assessment system in a murine model of spinal cord injury. We used image processing and 3D computer vision techniques to automatically calculate and track individual animal’s CoV position in the same open-field environment currently used for visual assessment. Parameters were identified that automatically classify movement type. To determine the relevance of the new outcome measures for SCI, we tested predictive validity with the underlying SCI neuropathology and concurrent validity with open field BMS recovery scores.

## Methods

### Ethics Statement

All procedures used in this study strictly adhered to the National Institutes of Health’s Guide for Care and Use of Laboratory Animals. The protocol was approved by The Ohio State University Institutional Laboratory Animal Care and Use Committee (2010A00000077). To minimize animal suffering, surgical procedures were conducted under ketamine and xylazine anesthesia.

### Experimental Methods and Data Processing Procedures

Three-dimensional movements of individual animals in an open-field environment were measured using markerless tracking and were used to automatically classify animal behaviors. Twenty animal’s trials were 2-minutes long, and two animals had slightly shorter trials. Eight naïve and 14 SCI mice (Jackson Laboratories C57BL/6J) were evaluated to develop parameters that identified differences in animal behavior and in motor function across injury severities. Midthoracic injury (T9) of the spinal cord was produced either by contusion, using the Infinite Horizon Device with forces between 60–90 kdynes, or by transecting the cord (for details see [30], [31]). Such injuries produced hindlimb deficits while movement of the forelimbs remained intact. The animals were evaluated 5–7 weeks post injury, ensuring a stable, plateaued performance.

Motor function capabilities of the mice were evaluated using the BMS, and BMS scores correlate highly with the extent of the neuropathology in the injured spinal cord across a range of severities [9]. Thus, we are able to grade the outcome of SCI according to BMS scores. Four groups were categorized: naïve (BMS score 9, n = 8), mild (BMS range 7–8; mean ± SD: 7.0±0, n = 5), moderate (BMS range 4–6; mean: 4.9±0.2; n = 5), or severe (BMS range 1–3; mean: 1.25±0.5, n = 4). In general, mice classified as mild walk with subtle gait deficits of trunk instability and paw rotation. Those in the moderate group step with the hindlimbs but not reliably and lack coordination between the forelimbs and hindlimbs. The severe mice do not support weight with the hindlimbs and produce only joint movements.

To quantify the extent of neuropathology at the injury site, mice were transcardially perfused with 0.1 M phosphate buffered saline (PBS; pH 7.4) followed by 4% paraformaldehyde (pH 7.4). A 10 mm block of spinal cord containing the lesion site was post-fixed for 1 h in 4% paraformaldehyde, rinsed overnight in 0.2 M phosphate buffer (PB, pH 7.4) then cryoprotected in 30% sucrose before being frozen on dry ice [9], [32]. The epicenter block was transversely sectioned at 20 um on a Microm HM505E cryostat and stained for myelin using eriochrome cyanine. The section with the largest lesion and least amount of stained white matter represented the lesion epicenter. Area of stained white matter at the epicenter was divided by the total cross sectional area of the uninjured cord at T9 and served as a measure of injury severity [13].

**Figure 1:**
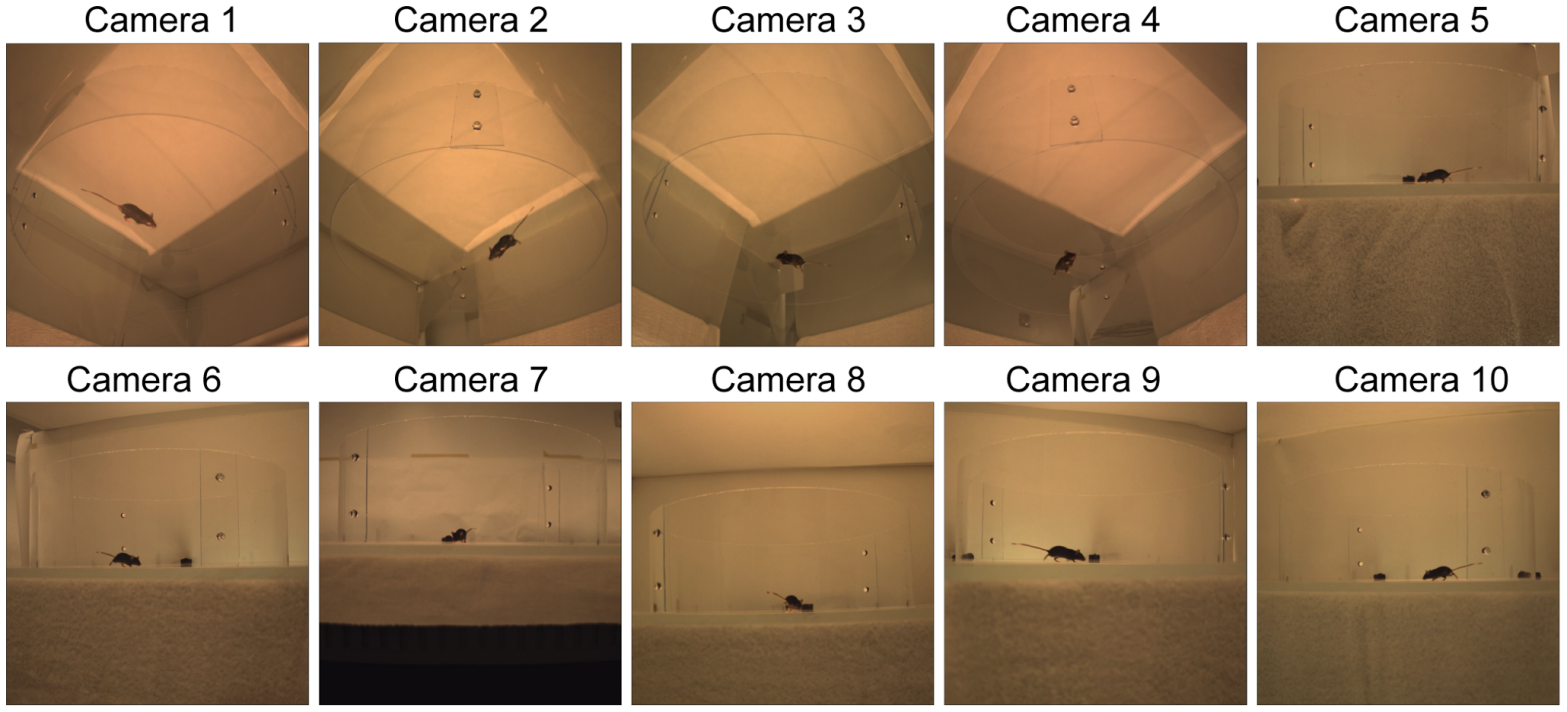
Synchronized images from each of the 10 cameras. Four cameras were positioned under the capture volume (1–4) and six parallel to the floor (5–10) to provide 10 unique silhouettes of the animal at each time.

Animal movements in the 36in diameter, round, open-field environment were captured using ten synchronized video cameras recording at 100 frames/s (Prosilica GX1050, 1024×1024 pixel resolution, custom data collection system designed and built by Spica Technology Corporation). The cameras were placed such that the entire area was visible in every camera view, and every point could be observed by at least two cameras (Figure 1). Six cameras were positioned with views parallel to the testing surface in order to clearly differentiate the limbs and torso from the ground (Figure 1, cameras 5–10). The floor of the open-field environment was made of clear acrylic, and the remaining four cameras viewed the animal from underneath to define the overall shape and foot position (cameras 1–4). The cameras were calibrated to remove image distortion and to define their 3D spatial relationship relative to the testing environment [33]. The video image streams were then analyzed to calculate the three-dimensional shape of the naïve and SCI mice.

**Figure 2:**
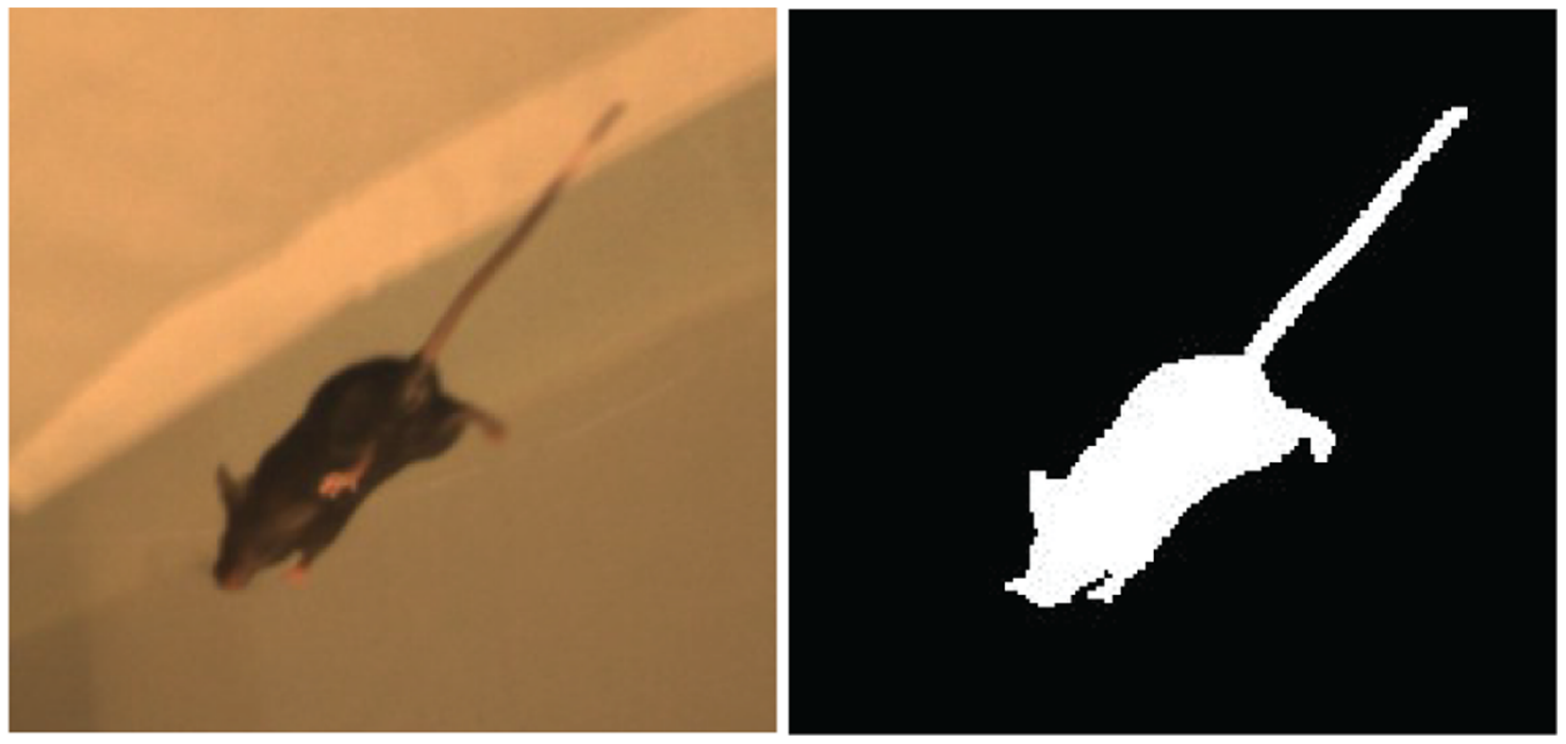
Image of a mouse (A) and silhouette of this mouse following background subtraction (B). Silhouettes from all 10 cameras were calculated and projected into the capture volume to define the 3D shape of the animal throughout the 2-minute, open-field trial.

Information about an animal’s shape was derived from its silhouette when viewed from a known camera perspective (Figure 2). By combining the silhouettes from multiple images recorded simultaneously from known perspectives, the animal’s shape and volume were calculated [20]. The 3D shape was reconstructed by first dividing the entire capture volume into a set of 2 mm×2 mm×2 mm cubes called voxels, and calculating which of these voxels corresponded to the animal by back-projecting each silhouette into the calibrated space. The animal surface was defined by the intersection of the projection cones (shape-from-silhouette), and the 3D volume of the animal was calculated by summing the set of interior voxels. The accuracy of the reconstructed shape increased with the number of silhouettes recorded from unique perspectives, which would enable a more accurate surface reconstruction, and the resolution of each image, which would increase the number of pixels associated with the animal and allow for a decreased voxel volume. By repeating this procedure for every video frame, the animal’s shape and global position throughout the trial was calculated.

If the animal’s silhouette was perfectly defined in each image, only the volume occupied by the animal would correspond to all silhouettes; therefore, the accuracy of the shape-from-silhouette approach [20] depended on the accuracy with which the animal’s silhouette was separated from the background. Separation was automatically performed by recording an image of the background without the animal, and then subtracting the RGB color value of each pixel in the background from images including the animal (Figure 2). After background subtraction, the RGB values were weighted and summed, to create an intensity image and any pixel that had a value exceeding a small threshold (to account for pixel noise) corresponded to the animal [34].

Accurately defining the animal’s silhouette was the most important step for ensuring data accuracy and repeatability. Background subtraction errors were caused by background color changes that were not associated with the animal and/or when the animal matched the background too closely; therefore, the background color during this experiment was carefully controlled. While testing black mice, a white environment was constructed around the open-field, and the background image was automatically processed to re-color the black camera lenses to be white. Any color animal could be tested as long as the background is a contrasting color. Additionally, data quality was improved by constructing a bounding cube around the animal, performing image dilation and erosion prior to analysis, and using a conditional probability model to identify pixels corresponding to the animal [26].

A bounding cube approach reduced errors caused by background changes far from the animal and decreased processing time [26]. A cube surrounding the animal was automatically identified in the first video frame, by subtracting the background for each pixel in each camera view. To process the next frame, the cube expanded in all directions and background subtraction was only performed for the pixels inside of this cube. The new animal CoV position was calculated, and the cube was re-centered so that it automatically moved with the animal in the capture volume. Background subtraction was the most time intensive video processing step because a separate calculation was performed for each pixel in each frame (1,048,576 pixels) and for each camera view (10 cameras). This bounding cube approach removed at least 80% of the image pixels to be processed and reduced processing time by an order of magnitude.

**Figure 3:**
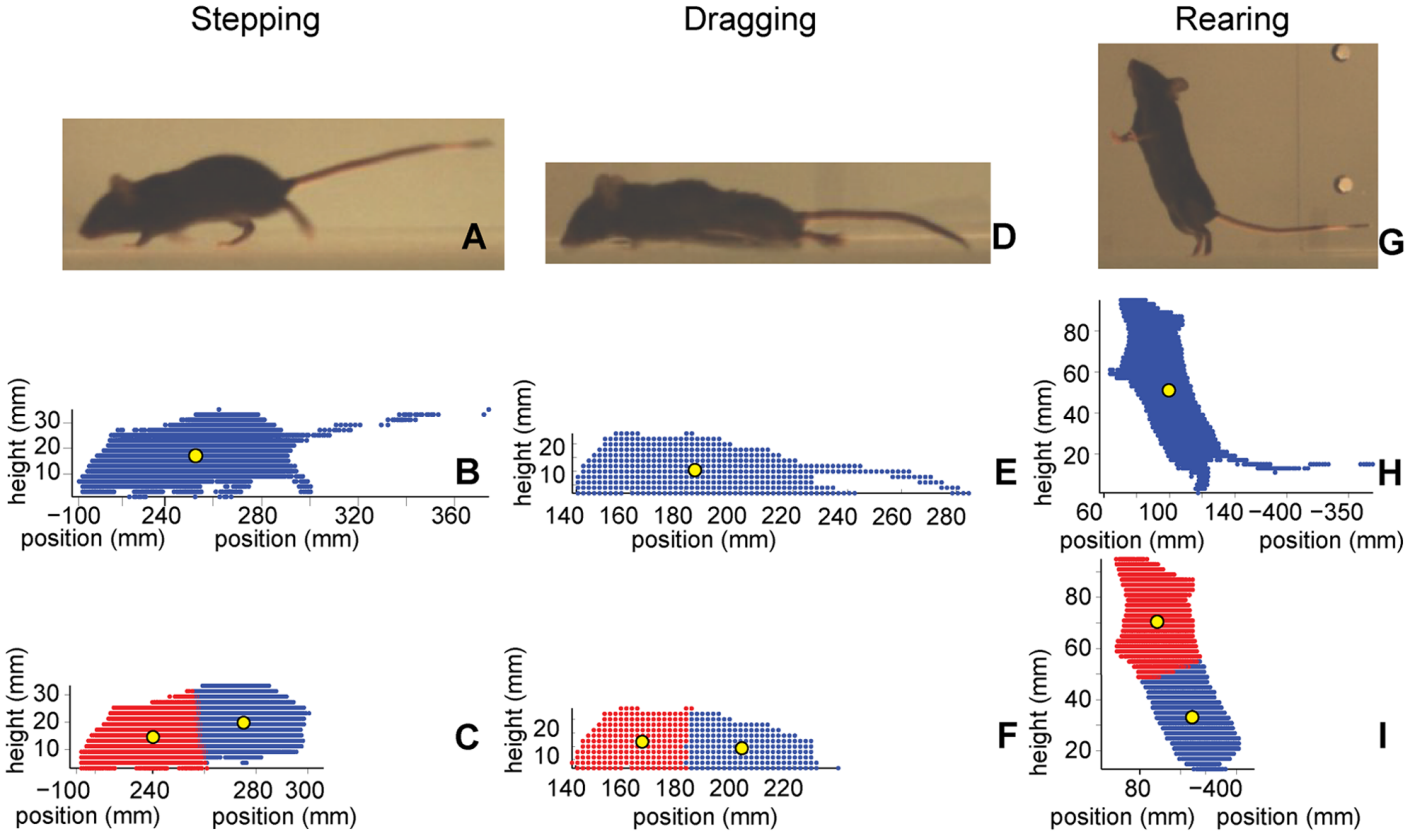
Video frames and corresponding reconstructed 3D volumes of whole and segmented mice. Representative video frames of directed locomotion (A), dragging (D) and rearing (G). The corresponding reconstructed 3D volumes of subjects including the tail (B, E, H), and divided into front and rear segments without the tail (C, F, I). The center of volume of the animal’s front and rear halves are used in the detailed analysis of directed locomotion bouts to reduce the influence of head motion.

Volume reconstruction errors within the bounding cube were reduced by removing small imperfections using morphological image processing (erosion and followed by dilation). Two separate threshold elements were used in the morphological image processing in order to reconstruct the entire 3D shape of the animal both with and without the tail (Figure 3). The tail was removed in order to reduce the influence of this highly mobile appendage on parameters derived to describe the gross position and shape of the animal body. To reconstruct the entire animal, isolated elements smaller than a square 3×3 pixel size were discarded. Elements smaller than 7×7 pixels were not considered to be part of the animal in order to remove the tail. For significantly larger or smaller animals, capture volumes, or camera resolutions, these values would need to be adjusted.

To further reduce volume reconstruction errors, the likelihood of whether or not an individual voxel in the capture volume belonged to the volume occupied by the animal was calculated given the total animal volume, number of camera correspondences, and the spatial continuity of the volume [26]. The volume of the animal (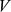) is the collection of voxels in the capture volume (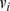) that exceed a likelihood threshold (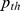)
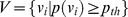
(1)where 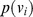 is the likelihood index of each voxel 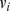. The likelihood index is the sum of the number of cameras that identify a particular voxel as belonging to the animal volume (correspondences), and the spatial distribution of the voxels associated with the animal [26].
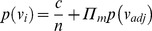
(2)where 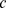 is the number of correspondences, 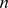 is the number of unique camera views (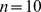), and 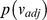 represents the initial indices of the 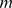 immediate adjacent voxels of 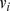. The threshold 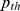 for each frame is defined such that the mouse’s estimated volume is within three standard deviations, 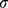, of the mean mouse volume, 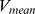 [26].



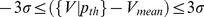
(3)If the total animal volume was greater or less than 3 times the standard deviation of the average mouse volume, the threshold was iterated (lowered) until this condition was met (Equation 3).

The reconstructed mouse shape without the tail was divided into a front and back half using k-means clustering in order to reduce the influence of head motion on the measurements used for evaluating movement quality (Figure 3). The mouse volume was specified to be composed of two clusters, and the points were optimally assigned to each cluster by minimizing the squared distance from each point to the cluster mean. Initially, three clusters were specified for segmenting the mouse volume with the tail, in an attempt to measure the position of the tail as well as the front and back of the animal. Consistent tail segmentation was not achieved due to the segment’s large range of motion.

Each animal’s shape and position throughout the trial was calculated by repeating the background subtraction, volume reconstruction, and front/rear segmentation procedure for every video frame. From these volumes, measures to describe the animal’s spatial position, speed and shape were defined and then used to broadly classify animal behaviors and evaluate motor function.

The center of volume (CoV) position of the front, back, and whole mouse were calculated by averaging the position of the center of each 3D voxel associated with the animal (Figure 3). Over the 2-minute trial, these positions were filtered forward and backward with a low-pass Butterworth filter with a 20 Hz cut-off frequency. Front, back, and whole body CoV velocities were calculated by fitting quintic splines to the filtered position data, and then differentiating the splines. The speed of each CoV was defined as the magnitude of the velocity in the plane of the open-field, and velocity in the vertical direction was not considered.

Occurrences of mouse ambulation, standing, and rearing were automatically identified during the two-minute trial in the open-field using the whole animal CoV speed and position, and front CoV height (Table 1). Ambulation was further classified as directed locomotion, exploratory locomotion, and meandering by considering the speed and distance the animal moved. Two expert BMS evaluators verified the accuracy of the automatically classified movements by watching videos of the 2-minute trials and identifying bouts that would be used to evaluate coordination (called directed locomotion in this manuscript), and bouts that were too short (exploratory motion). The timing of these bouts was recorded and compared to the automatic classification.

**Table 1:** Definitions used for automatic classification of movements in the open-field.

Broad movementclassification	Specific classification	CoV Speed	CoV Distance	Front CoV Height
Ambulation	Directed locomotion	≥60 mm/s without slowingfor more than 0.17 s	≥200 mm	<34 mm
	Exploratory locomotion	≥60 mm/s	<200 mm	<34 mm
	Meandering	≥10 mm/s and <60 mm/s	n/a	<34 mm
Standing		<10 mm/s	n/a	<34 mm
Rearing		n/a	n/a	≥34 mm

Discrepancies between the computer-generated classification and BMS evaluators either emphasized the repeatability of the automated approach or were incorporated into the classification algorithm and improved robustness. The algorithm was able to detect whether the locomotor bout was long enough when very close to the minimum 200 mm distance threshold (roughly 3 times the mouse body length) more consistently than the raters. Also, the comparison highlighted the need to allow for small breaks in speed during directed locomotion. In SCI animals, these slower locomotion periods were very difficult to notice by visual observation alone. Raters tolerated brakes in speed up to 0.17 s long in the SCI animals, and viewed these bouts as continuous yet with some obvious impairment while the algorithm separated the movements. When the video was reviewed and focused on select intervals, the BMS evaluators were able to identify the short periods of slower locomotion. Many times this speed change was due to a missed step, lateral instability, or a muscle spasm. Since the naïve animals typically moved with a faster speed, any slowing to speeds <60 mm/s were easily observable. Within this 0.17 s window, naïve animals could go from fast walking to stopped and return to fast walking, and this break in speed typically corresponded to the animal moving its head or sniffing. For consistency within this experiment, the definition of directed locomotion was developed for SCI animals and applied to naïve animals.

The definition used for automatic classification of directed locomotion was when an animal’s whole body CoV moved a distance of at least 200 mm without dropping below a speed of 60 mm/s for longer than 0.17 s at a time (Table 1). The definition for directed locomotion allowed for walking slower than 60 mm/s for periods shorter than 0.17 s in order to better align with bouts used to identify coordination by the BMS raters for the SCI animals. During exploratory locomotion, an animal moved faster than 60 mm/s for a distance shorter than 200 mm before slowing for longer than 0.17 s (Table 1). All movements faster than 10 mm/s and slower than 60 mm/s, that were not associated with directed locomotion, were classified as meandering regardless of distance covered. Standing occurred when the animal speed was slower than 10 mm/s, and the height of the front CoV position was shorter than 34 mm. The front CoV position was taller than 34 mm when rearing (Figures 3g–i).

Mouse movements over the two-minute open-field collection period reflect both motor capabilities and behavioral preferences. In our experience with SCI, directed locomotion bouts primarily reflected motor capacity while behavioral influences were minimized. Exploratory locomotion and meandering (i.e. shorter and/or slower walking bouts) were also influenced by motor capacity, but have a much larger influence from behavioral preferences. Therefore, to investigate each animal’s motor function capabilities, directed locomotion bouts were identified, and CoV height, speed, and lateral deviation were calculated.

**Figure 4:**
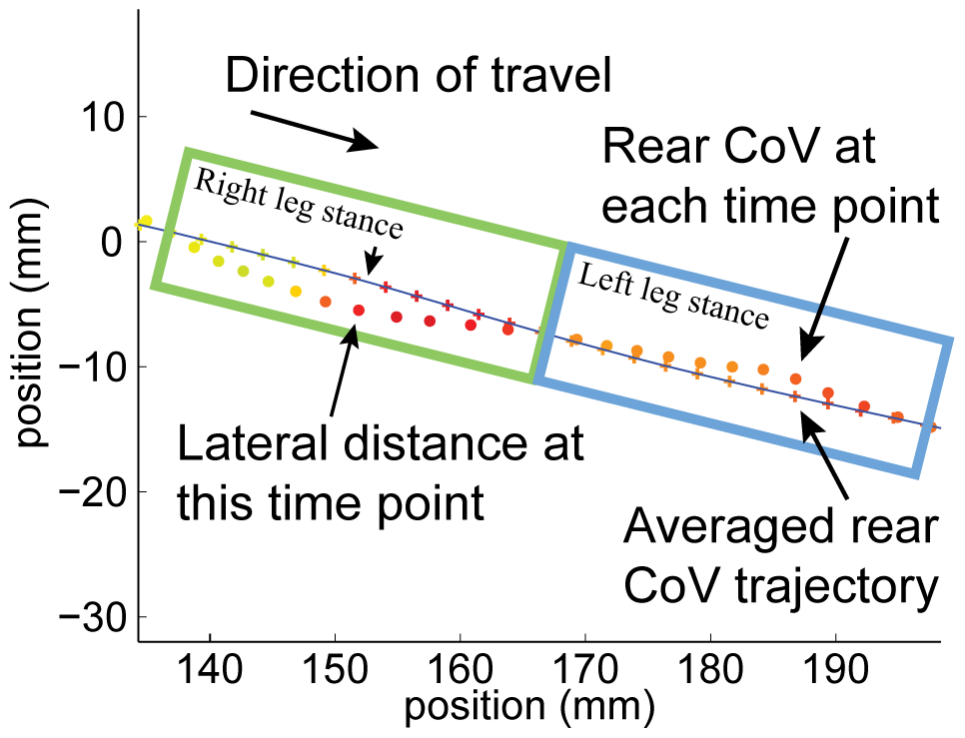
Top-down view of the lateral deviation of the rear CoV during directed locomotion. A top-down view of the rear CoV position in the testing plane at successive points in time (circles, each point is 0.01 s apart) and an averaged rear CoV trajectory (blue line) is shown over one-step cycle lasting 0.24 s. The rear CoV lateral deviation was calculated as the perpendicular distance of the rear CoV position at each point in time from an averaged rear CoV trajectory.

Lateral deviation of the front and rear CoV were defined as the perpendicular distance of each instantaneous CoV position from the corresponding points in an averaged movement trajectory (Figure 4). The averaged trajectory was calculated using a central moving average of the filtered CoV positions. The number of points used in the moving average was a function of the speed of the animal- fewer points were used for faster moving animals, and more points were used for slower moving animals. Since lateral deviations frequently accompany weight transfer during stepping, in order to not bias the averaged trajectory to the left or right of the animal, the number of points included in the moving average corresponded to a single step cycle (a single step with each of the right and left limbs). A relationship between speed and step length was developed to determine the number of points used in the moving average.

In order to develop the relationship between speed and step length, the Euclidean distance moved by the rear CoV for each step cycle (i.e. one step of each hindlimb in higher functioning animals, or forelimb if dragging) during each directed locomotion bout was calculated. Individual steps during each bout were automatically identified by finding local minima and maxima of the lateral deviation of the rear CoV relative to the averaged trajectory (Figure 4). In order to reduce step-to-step variation or small errors in the data, an average distance per step for each directed locomotion bout was calculated by dividing the total distance moved in the bout by the total number of steps. The average speed for this directed locomotion bout was also calculated. Each directed locomotion bout from each animal contributed a single point to the speed and step length relationship.

Initially, 31 points (the value at the specified time and 15 before and after) were included in the central moving average. A linear relationship between directed locomotion speed and distance per step cycle for all animals was observed and quantified, and this relationship defined the number of points to be included in the moving average. The rear center of volume position data was then reprocessed and for each moment in time during the 2-min data collection, the number of points included in the moving average was calculated using the linear regression equation relating speed (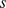) to the distance traveled in one step cycle of the left and right hindlimb (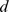).
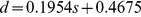
(4)


This distance was then divided by the speed, multiplied by data collection frame rate (100 frames/second), and rounded to the nearest whole integer to define the number of frames considered in the moving average. For the slowest moving animals, a maximum of 61 frames (30 before and after) were included in the moving average. This process was repeated and the linear relationship was iteratively re-calculated until the fit of the linear regression equation changed by less than 1% (Equation 4).

### Statistical Analysis

The primary outcome measures of speed and rear CoV height averaged across all directed locomotor bouts during each 2-minute collection period were tested for predictive and concurrent validity using Pearson Correlations. The relationship of these outcome variables to the percentage of white matter sparing at the epicenter (predictive) or to BMS scores (concurrent) for mice with SCI (n = 14) were derived.

## Results

**Figure 5:**
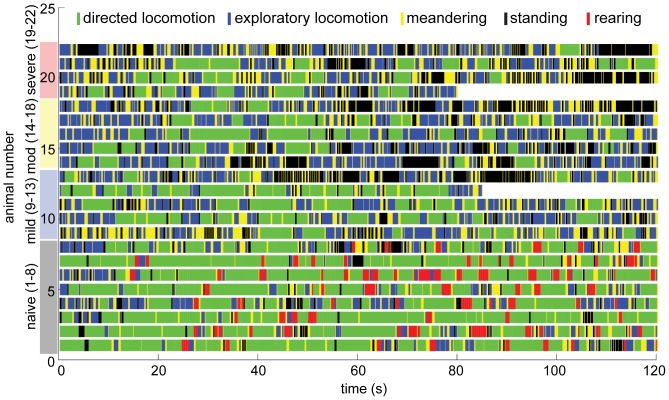
Automatically classified behavior for each animal throughout the 2-minute long trial. Animals spent more time standing and rearing during the second half of the trial, and only naïve animals reared during this experiment.

Animal movements throughout the 2-min duration, open-field trial were automatically classified using front CoV height and whole body CoV speed, which were calculated from the reconstructed 3D shapes (Figure 3). All animals displayed periods of directed locomotion (Figure 5, green), exploratory locomotion (blue), meandering (yellow), and standing (black) during the 2-minute trial. Only the naïve animals reared (Figure 5, red). In order to separate movement parameters that could be more influenced by behavioral preferences from those that more closely reflect motor control strategies and functional impairments, directed locomotion bouts were isolated, and analyzed.

**Figure 6:**
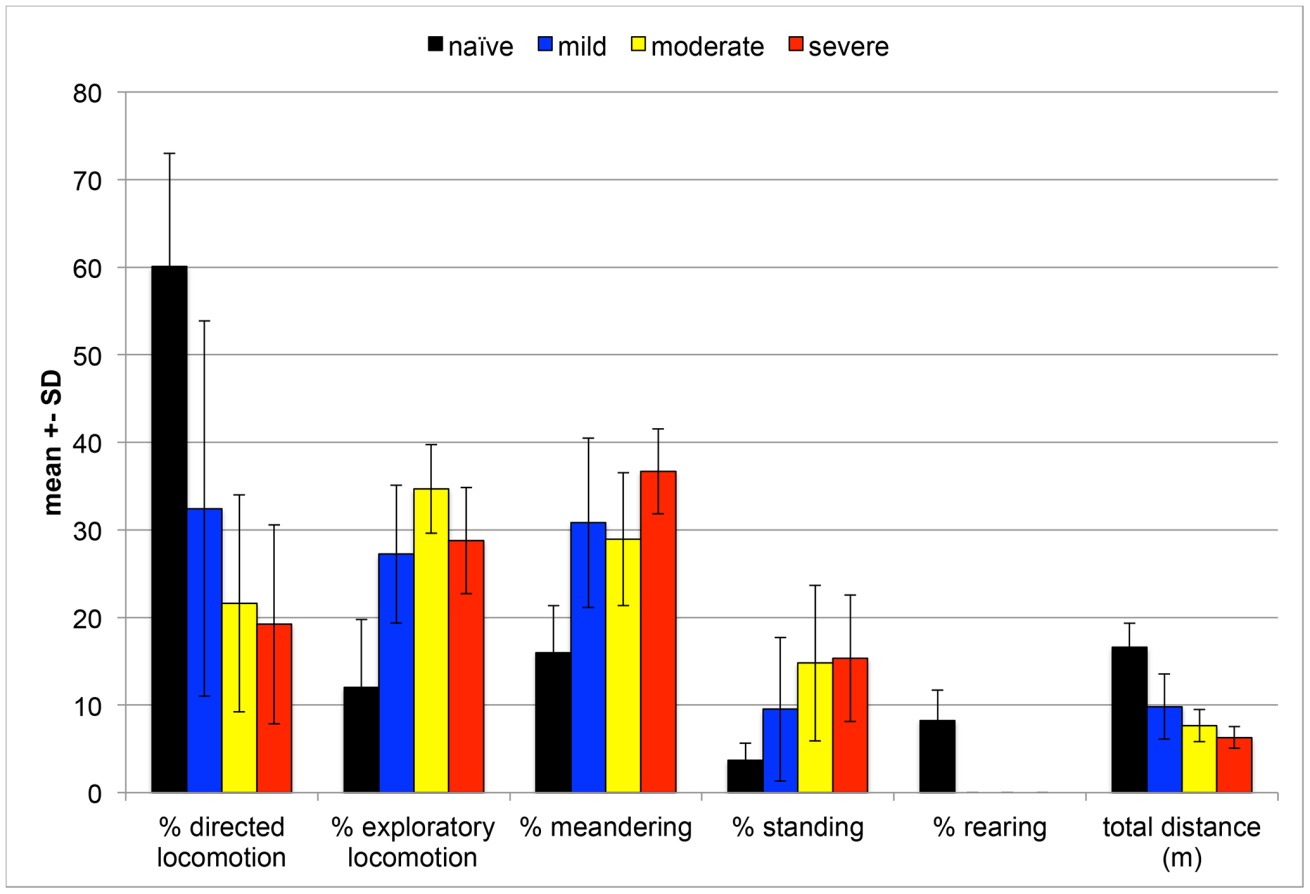
Percentage of trial each group spent performing classified behaviors and total distance covered. Mean and standard deviation of the percentage of the two-minute trial spent performing each movement and the total distance moved during the trial. Naïve animals spent the largest percentage of the trial in directed locomotion, and the least standing.

During the 2-minute trial in the open-field, the naïve animal had numerous clearly defined bouts of directed locomotion (Figure 5, green) separated by short periods of standing and/or rearing (black and red). As a group, the naïve animals spent the most time performing directed locomotion, walked the farthest distance at the fastest speeds, and spent the least amount of time meandering and standing (Figure 6). As SCI severity increased, the total distance traveled decreased and time spent meandering increased. All animals spent a larger amount of time performing directed locomotion during the first half of the trial, and more time standing and rearing during the second half of the trial (Figure 5).

**Figure 7:**
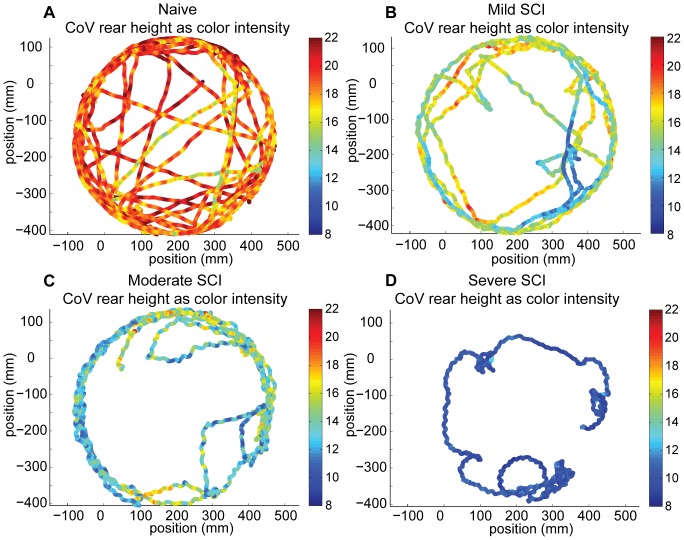
Top-down view of movement paths of a naïve and 3 SCI mice of different severities. These animals are representative of the distance traveled in an open-field trial for each group. Each dot represents the position of the mouse rear CoV in the open-field environment at 0.01 s intervals during the 2-min long trial. The color of the dot indicates the height of the rear CoV, and the rear CoV was highest (denoted by red) for the naïve mouse and lowest (denoted by blue) for severe SCI.

**Figure 8:**
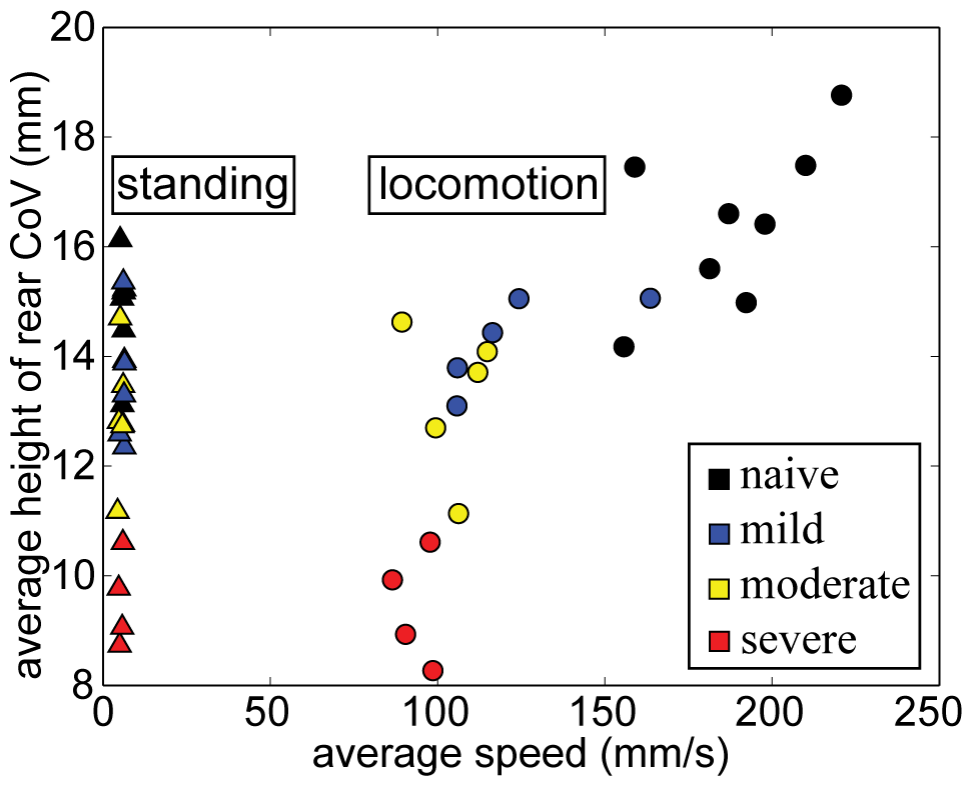
Average rear CoV height vs. average speed during standing and locomotion faster than 60/s. Both directed and exploratory locomotion are included in these data, as long as the animal’s speed was faster than 60 mm/s. Considering speed and rear CoV height in combination distinguished naïve from moderate and severe SCI groups, and mild from severe SCI.

The trajectory of the rear CoV in the plane of the open-field environment during the two-minute trial revealed behavioral and kinematic differences between naïve and SCI animals (Figure 7). Naïve animals showed the behavioral preference for crossing the center of the open-field multiple times, whereas mice with SCI had fewer center crossings and instead walked near the walled circumference. These behavioral differences between naïve and SCI were reflected across all animals tested even though each animal’s spatial and temporal distribution of movements was different. Independent of the distribution of movements, the average rear CoV height of the naïve animals was taller than that of the SCI animals, and the severe SCI animal’s average rear CoV height was shortest (Figures 7, 8). Also as SCI severity increased, lateral oscillations in the path of the rear CoV during ambulation emerged, the number of the lateral oscillations within a defined distance increased, and small areas with numerous tight turns appeared (Figure 7). The naïve animals walked with the straightest trajectories.

We quantified these findings by comparing each mouse’s average rear CoV height and average speed during standing, and during directed and exploratory locomotion (Figure 8). Naïve animals had the tallest rear CoV during locomotion faster than 60 mm/s, and the fastest average locomotion speed over the duration of the trial. Severe SCI animals had the shortest rear CoV height when standing or moving faster than 60 mm/s. Average rear CoV height for naïve mice was lower while standing than during ambulation, while the rear CoV heights for severe SCI mice were roughly equal. Differences between the mild and moderate mice rear CoV height were small, and there was no clear separation between these groups using only height and average locomotion speed.

We correlated the automatically-generated kinematic metrics with the extent of neuropathology of the injury and with the gold standard measure of recovery after SCI to test validity. In SCI, the severity of motor impairments coincides with the size of the neural injury [8], [9]. Greater lesion size results in poorer motor performance. Average speed and rear CoV height across all locomotor bouts was significantly correlated with the percent of white matter sparing at the lesion epicenter (speed r = .70 p<.005; rear CoV height r = .63 p<.01). Moreover, rear CoV height and speed were correlated with higher BMS scores (speed r = .72 p = .001; rear CoV height r = .86 p<.001).

**Figure 9:**
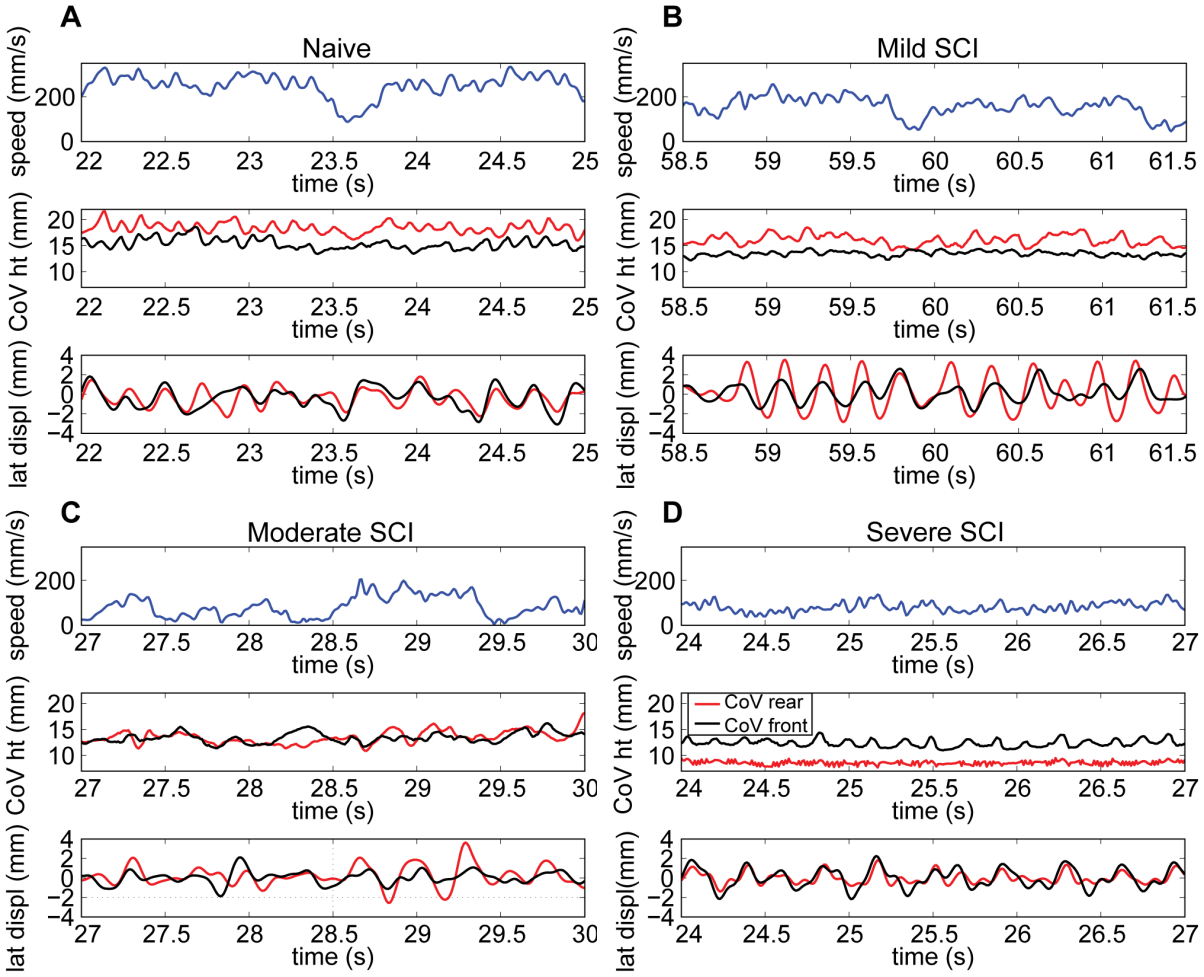
An animal’s locomotion speed, CoV heights and lateral displacements reflect motor SCI severity. Locomotion speed, height of the rear CoV (red) and front CoV (black) and lateral displacement of the rear and front CoV for a representative A) Naïve B) Mild SCI C) Moderate SCI D) Severe SCI animal.

Analysis of individual bouts of directed locomotion revealed differences in mouse motor function capabilities. Naïve animals had faster steady-state speeds, and higher rear CoV during bouts of directed locomotion when compared with SCI animals (Figure 9). Additionally, the relative height of front and rear CoV indicated SCI severity. Naïve animals rear CoV were higher than front CoV during almost all directed locomotion bouts (Figures 3a–c and 9, height 16–21.6 mm). When front CoV were higher, Naïve animals were typically moving more slowly with the nose up in the air, exploring the environment. For all animals, the front CoV was almost always taller than the rear when standing, or performing slow-speed exploratory movements. After SCI, rear CoV was incrementally lower as severity increased and hindlimb motor control worsened. However, there was little change in front CoV height. For mild SCI, the absolute height of the rear CoV was lower than that of Naïve animals (14.1–18.5 mm), and the rear CoV remained slightly higher than the front CoV during locomotion (Figure 9, mild). For most mice with moderate SCI, rear CoV height decreased further (10.9–16.1 mm) and was roughly equivalent to front CoV height. For severe SCI, rear CoV height was lowest of all groups (7.7–9.5 mm) and well below the front CoV position. Given the low position and lack of oscillation, the rear CoV for the severe group indicated dragging of the hindlimbs and lack of stepping.

The decreased motor control capability after SCI is also apparent in the larger deviation of the lateral rear CoV displacement. In naïve animals, the lateral deviation of the front and rear CoV mirror one another in phase and amplitude (Figure 9, naïve). As SCI severity increased, the number of steps taken decreased, and lateral deviation became greater during stepping and was much smaller in periods of no stepping. After mild SCI, hindlimb stepping was consistent but lateral deviation of the rear CoV exceeded that of the front CoV. For moderate SCI, hindlimb stepping was inconsistent. In this example, only three steps occurred with the right foot while dragging the left (Figure 9, moderate, time 28.5–29.5 s). These hindlimb steps are indicated by two factors: the three largest local maxima and minima in the rear CoV lateral deviation, and when the rear CoV was higher than the front CoV during directed locomotion. During locomotion when the hindlimbs drag, the lateral deviation of the front CoV is slightly larger than that of the rear because propulsion is being provided by the front limbs, as shown in severe SCI (Figure 9, severe).

**Figure 10:**
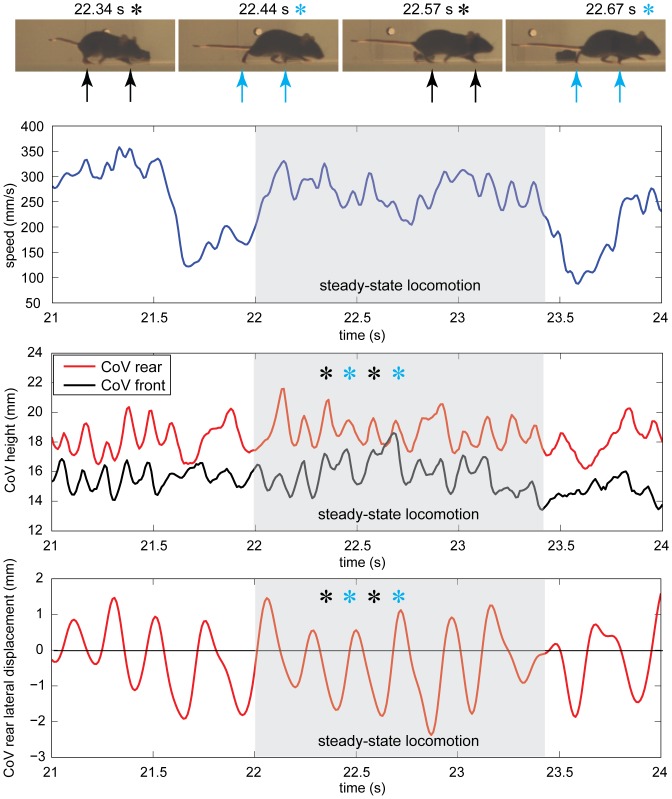
Patterns in the rear CoV height and lateral displacement indicate steps during steady-state, directed locomotion. Local maxima in the rear CoV height correspond to mid-stance during coordinated, steady-state locomotion in a naïve mouse. Lateral displacement of the rear CoV also corresponds to individual steps in naïve animals during steady-state locomotion and reflects the transfer of weight onto the stance leg.

Hindlimb stepping was indicated by local maxima and minima in the rear CoV height and rear CoV lateral deviation during steady-speed portions of directed locomotion for the naïve animals. Peaks in the rear CoV height corresponded to mid-stance for each of the hindlimbs; therefore, every other peak corresponded to mid-stance of the right hindlimb, and alternate peaks to the left hindlimb (Figure 10). A similar pattern in the front CoV was observed during faster walking (roughly >100 mm/s) and corresponded to forelimb stepping. A consistent timing offset between the front and rear local maxima were observed during manually identified periods of constant-speed, coordinated (diagonal) gait (Figure 10). Local minima and maxima in the lateral deviation of the rear CoV also indicated rear limb steps. Local maxima were produced during the right hindlimb swing phase when the animal shifted the weight left to clear the right toe, and local minima occurred when the left toe cleared (Figure 10). The magnitudes of the local minima are slightly larger than the magnitudes of the maxima because the animal was walking in a counter-clockwise arc around the edge of the round open-field environment. When the animal is walking in an arc, the moving average position would be slightly biased toward the inside of the curve.

The temporal pattern of peaks in the rear CoV height and lateral deviation plots are disrupted during the acceleration and deceleration periods as the animal moves in and out of steady-state walking speed (Figure 10, just before and after the shaded region). During these periods, movements of the forelimb and hindlimbs were not coordinated. Also, during deceleration the mouse frequently moved its head upward. Since the head is a large percentage of the volume of the front half of the body, head movements decreased the influence of the front leg movement on the front CoV position.

**Figure 11:**
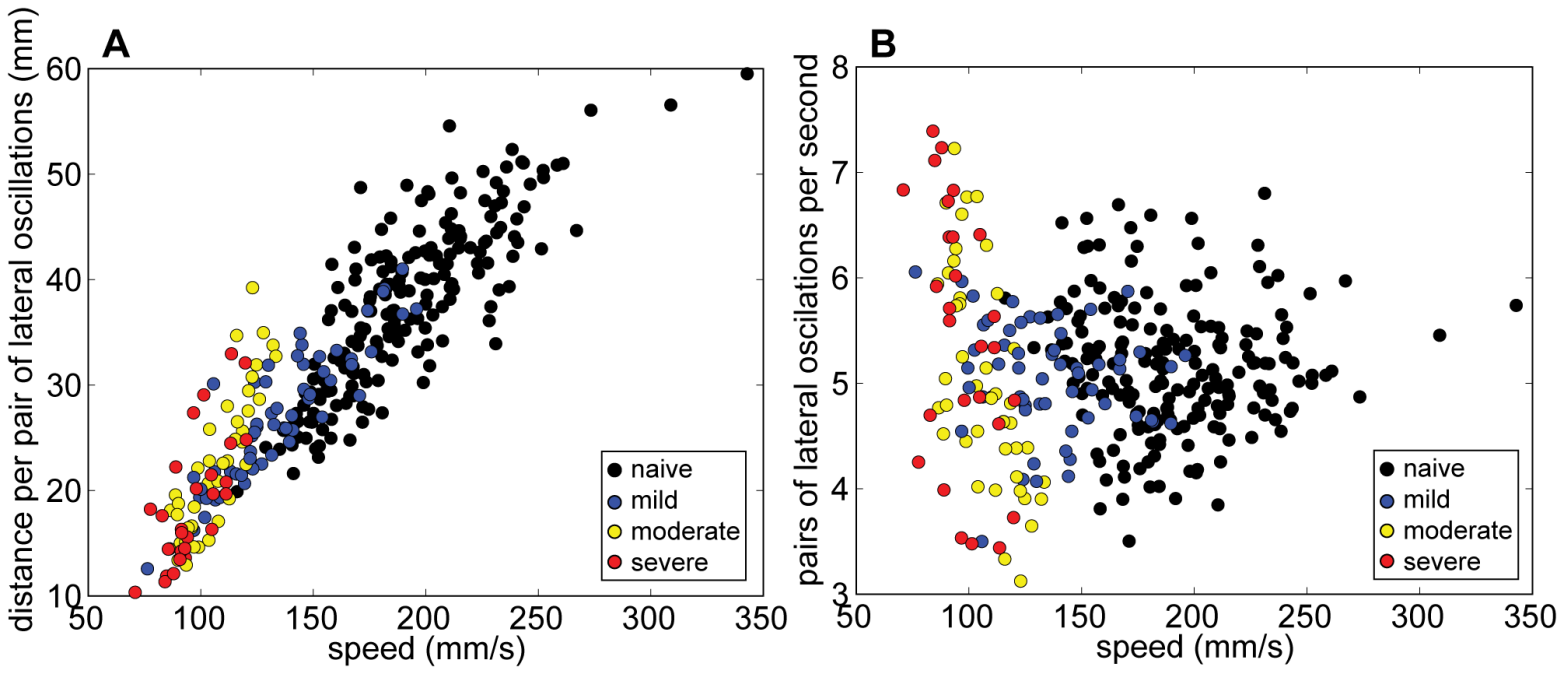
Relationship between walking speed, step length, and step frequency. A) When considering all walking bouts, mouse step length, calculated from lateral oscillations of the rear CoV, increased with walking speed. B) No clear relationship between step cadence, calculated from the rear CoV lateral oscillations, and walking speed was observed.

Locomotion speed is a function of step length and step cadence. During directed locomotion, oscillations in the path of the rear CoV corresponded to stepping regardless of whether an animal used the hindlimbs (all naïve and mild SCI animals) or forelimbs (all severe SCI animals) for propulsion (Figure 7, 10). For each locomotion bout for each animal, the average speed was plotted as a function of the average distance traveled per lateral oscillation (i.e. step length), and also as a function of the average lateral oscillation per second (i.e. step cadence). Mice appeared to walk faster by taking longer steps, rather than by drastically increasing step rate (Figure 11). It is possible that mice with moderate and severe SCI could not walk as quickly as naïve and mild SCI animals due to motor deficits that affected the ability to increase step length (i.e. range of motion) or to develop propulsive forces.

## Discussion

Using markerless motion tracking, mouse behavior and movements were automatically classified and analyzed in the open-field to determine functional impairments after SCI. Large differences were found between naïve mice and SCI mice. Naïve animals displayed the widest variety of behaviors including rearing, walking at speeds of 60 mm/s to faster than 340 mm/s, and positioning the rear CoV at heights from 10 mm to greater than 30 mm. SCI reduced the range of behaviors and speeds the animal achieved, and lowered the rear CoV height. Importantly, these automatically-measured reductions in motor function were associated with greater lesion size, thereby showing evidence of predictive validity.

Analysis of open-field performance for a sustained period of time exposed SCI-induced impairments that might not have been apparent with constrained locomotion such as on a walkway. Clear behavioral differences emerged; namely that SCI mice no longer reared and avoided crossing the center of the open-field. Additionally, mice spent different portions of the 2-minute trial performing directed locomotion, exploratory locomotion, meandering and standing, which generally aligned with injury severity. In this experiment, greater SCI severity resulted in a decreased total locomotion distance in the open-field environment. In-depth analysis of temporal and spatial variation during directed locomotion further delineated the motor capacity of naïve and neurologically impaired mice.

Whole body, front and rear CoV positions and speed not only classified types of behavior but also differentiated severity of motor impairments. These measures emerged out of all that could be calculated from an animal’s 3D shape because they have biological significance as well as the ability to differentiate injury severity. Height of the rear CoV separated high performing walkers that stepped from those with complete hindlimb paralysis (Figure 8, naïve 16.4 mm average height vs. severe SCI 9.4 mm). When combined with speed, impairments from mild, moderate and severe SCI were further delineated from normal (Figure 8). While rear CoV height and average speed demonstrated good concurrent validity with BMS scores, a limitation is that these kinematic parameters did not differentiate mild SCI from moderate SCI. With these just metrics, the moderate group displayed wide variation, which overlapped performances by the mild group. Few or no other assessment tools provide speed and CoV height as performance measures. The fact that both of these measures are associated with the amount of neural sparing at the lesion site and can be used to describe behavior also make them innovative.

More detailed analysis of the lateral position of the rear CoV also provided kinematic metrics of walking including step cadence, stride length and balance/instability. That individual steps could be identified during steady-speed directed locomotion by the front CoV and the rear CoV may allow us to develop a metric of forelimb-hindlimb coordination, an important benchmark of recovery after SCI. Multivariate analyses are underway to determine if a combination of parameters may distinguish differences in motor capacity for the mild and moderate groups.

The markerless tracking approach combines the quantitative information available using existing commercial products with the behavioral benefits of performing experiments in an open-field environment. Current methods of evaluating mouse motor capacity primarily focus on open-field behavior using measures of activity or walking performance using standardized visual observation (BMS), or kinematics collected via camera systems for walkway or treadmill constant speed movement (CatWalk by Noldus Information Technology, TreadScan by Clever Sys Incorporated, DigiGait by Mouse Specifics, Inc). The limitation of these approaches is that none provide both quantitative and qualitative assessments of movement.

The markerless motion capture approach appears to provide qualitative measures of behavior and movement concurrently with kinematic metrics. The system provides data on types of movement much like an activity box while producing stepping metrics like CatWalk (Noldus Information Technology) or kinematics. Unlike quantitative techniques of the CatWalk (Noldus Information Technology) and kinematics, this new approach can be used across the full range of motor performance from normal walking to paralysis and all data processing is automated. In mice, only the BMS applies across this broad range but it relies on subjective observation, which introduces variability from lab to lab. Although the BMS is used as a benchmark for the concurrent validity portion of this experiment, the markerless motion capture method improves upon the BMS by reducing subjectivity, and enabling the consideration of additional information that cannot be detected by observation alone. This may allow the new method to be more sensitive than the BMS in the future. Additionally, the markerless approach allows for the analysis of numerous locomotion bouts for an individual animal with different speeds, lengths, and movement directions. This is an advantage because movement variability within an animal is affected by SCI, and including more than a single type of directed locomotion bout for evaluating an animal’s motor function abilities enabled larger separation between the SCI groups than only considering their longest walking bout (i.e. a measure of their “best” locomotion performance). Thus, markerless motion capture appears to combine the strengths of current analysis tools while addressing their weaknesses.

Limitations of this markerless method are that it cannot currently differentiate dorsal from plantar foot stepping, and only a limited range of behaviors can be automatically identified, fewer than a trained human observer. For example, secondary behavioral complications such as bowel/bladder effects, spasms, and scoliosis are not identified in this analysis. Although these secondary effects can give insight into the animal’s functional capability, results from this experiment indicate that these additional movements are not necessary to predict an animal’s underlying lesion size.

To improve the utility of the markerless motion capture assessment, outcomes from this experiment will be used to minimize trial length, and the number of cameras required to produce the behavioral and kinematic metrics of open-field performance. For example, during this experiment all animals, regardless of locomotor capabilities, were more active during the first minute of testing than the second. More directed locomotion occurred in the first minute and a greater tendency toward non-movement (standing and rearing) occurred in the second minute (Figure 5). Considering only the first minute of data collection also had little impact on distribution of total walking time. This indicates that trials may be shorter than 2-min while maintaining sufficient exemplars of behavioral influence and motor control capabilities. Shortening the trial length will reduce personnel time needed for testing, enable more animals to be tested, reduce data storage needs, and reduce the automated processing time. We expect further reductions in data collection complexity, storage, processing and cost by decreasing the number of cameras needed to accurately reconstruct the animal’s 3D shape. Accuracy is affected by the number of pixels corresponding to the animal, and the number of unique animal silhouettes. In this experiment, each video was approximately 1.2 GB for a 2 min trial, and unattended processing time on a standard MacBook air was approximately 12 hours per trial. Minimal attempts have been made to increase the efficiency of the code, and simply by multi-threading the current algorithm this time would be significantly reduced. Decreasing processing time has not been a priority because this step is fully automated, and can be left to run unattended or while pursuing other work.

With continued development, markerless motion capture would allow investigators to include more animals, perform more evaluations over the treatment course and pre-screen animals for motor dysfunction without lengthening total testing time. This markerless tracking and automatic movement classification approach, which was effective in mice with SCI, would likely generalize to larger rodents or other animals with motor deficits, since they are larger, move slower, and display a smaller variety of movement deficits. Additionally, this approach could be applied to rodent models for other diseases, for genetic mutation screening, and for identifying animals with behavioral differences.

The markerless motion tracking approach presented here is a first step toward fundamentally changing how rodent movement studies are conducted. By providing scientists with methods to collect and automatically process sensitive, quantitative data, the greatest barrier to assessing motor capacity accurately will fall. By being automated, our approach removes the requirement for biomechanically trained personnel and the huge time burden of analysis for experimental labs. Furthermore, markerless motion tracking can be used in the open-field environment for highly functioning animals as well as those with large motor deficits. With the development of more sensitive, repeatable and efficient tools for quantifying all animal motions, researchers can gain new insights into the mechanistic pathways of recovery in mice with musculoskeletal or neural injuries. Perhaps more importantly, scientists will gain tools to accurately evaluate new pharmaceutical interventions. Together, these insights could lead to breakthroughs in treatments of human disease.
